# The Impact of Low Serum Magnesium Levels on COVID-19 Severity and Potential Therapeutic Benefits of Magnesium Supplementation: A Systematic Review

**DOI:** 10.7759/cureus.77118

**Published:** 2025-01-08

**Authors:** Mehrab Hasan Majumder, Sadman Sazzad, Rabeya Hasin, Tasnim Jabbar Brishti, Fateha Nadia Tabassum, Tanvir Ahamed, Abdullah A Masud, Fahima Akter

**Affiliations:** 1 Internal Medicine, Bangabandhu Sheikh Mujib Medical University, Dhaka, BGD; 2 Health Informatics, Indiana University Indianapolis, Indianapolis, USA; 3 Medicine, Dhaka Medical College and Hospital, Dhaka, BGD; 4 Medicine, Ibrahim Medical College, Dhaka, BGD; 5 Gastroenterology, Bangabandhu Sheikh Mujib Medical University, Dhaka, BGD; 6 Medicine, Army Medical College Cumilla, Cumilla, BGD; 7 Internal Medicine, Saint Louis University School of Medicine, St. Louis, USA; 8 Internal Medicine, Indiana University Health Ball Memorial Hospital, Muncie, USA

**Keywords:** covid-19, hypomagnesemia, micronutrient supplementation, serum magnesium, severity of disease

## Abstract

In this review, our objective was to analyze the association between serum magnesium (Mg) levels, Mg supplementation, and coronavirus disease 2019 (COVID-19) outcomes. This systematic review followed Preferred Reporting Items for Systematic Reviews and Meta-Analyses (PRISMA) guidelines, searching major databases until February 2023. Twenty-six studies (11,363 patients) were included: 22 examining serum Mg levels (8474 patients) and four investigating Mg supplementations (2889 patients).

Most studies indicated an association between lower serum Mg levels and increased COVID-19 severity, including higher mortality rates and prolonged recovery periods. Critical patients demonstrated significantly lower Mg levels compared to moderate/severe cases. However, some studies reported conflicting findings, with hypermagnesemia also associated with poor outcomes in specific patient populations. Regarding supplementation, higher dietary Mg intake correlated with shorter hospitalization duration and faster recovery. Mg supplementation exceeding 450 mg showed potential benefits, including increased antibody titers in pregnant women and reduced oxygen support requirements in elderly patients when combined with vitamins D and B12.

While evidence suggests a potential relationship between Mg status and COVID-19 outcomes, findings are heterogeneous. Further investigation through well-designed clinical trials is required to gain deeper insights into the role of Mg in COVID-19 pathophysiology and the therapeutic potential of Mg supplementation.

## Introduction and background

Coronavirus disease 2019 (COVID-19) is an infectious disease caused by the severe acute respiratory syndrome coronavirus 2 (SARS-CoV-2) [1,2]. It emerged in Wuhan, China in 2019 and began spreading within human populations in the early months of 2020, causing a global pandemic. The SARS-CoV-2 virus can infect a wide range of cells and systems of the body. This virus accesses host cells via the receptor for angiotensin-converting enzyme 2 (ACE-2), which is most abundant on the surface of type 2 alveolar cells of the lungs. The emergence of the pandemic prompted the scientific and medical communities to take action. Their efforts focused on limiting viral transmission by identifying risk factors associated with poor disease outcomes [3,4]. Globally, as of August 9, 2023, there have been 769,369,823 confirmed cases of COVID-19, including 6,954,336 deaths [5].

Following the emergence of SARS-CoV-2, extensive research efforts have focused on elucidating the virus's pathophysiological mechanisms, particularly its cellular tropism, host-pathogen interactions, and molecular infection pathways. Of particular interest has been the virus's interaction with the ACE-2 receptor and subsequent cellular responses, which has profound implications for understanding disease progression and potential therapeutic interventions. Essential minerals play crucial roles in immune function and disease resistance. During the COVID-19 pandemic, researchers identified various mineral deficiencies as possible risk factors for severe disease outcomes.

Magnesium (Mg) emerged as particularly significant among these minerals due to its fundamental role in immune system regulation and inflammatory responses. When the pandemic began in early 2020, the global magnesium researcher group (MaGNet)[6] was alerted by the striking similarities between COVID-19 risk factors and conditions linked to Mg deficit state in humans [6-9], such as age, diabetes, obesity, high blood pressure, arrhythmias, thrombosis, and cardiovascular diseases. These conditions are associated with high odds of COVID-19 mortality [10,11], and they are common in Western societies and are spreading globally. Indeed, food processing losses result in lower dietary Mg intakes, and reduced Mg availability in the soil and consequently in the food chain, and long-term prescribed drugs such as proton pump inhibitors might precipitate subclinical Mg deficiency [12].

Our main area of interest has been the role of serum Mg levels in COVID-19 outcomes and the potential therapeutic benefits of Mg supplementation in COVID-19 patients. At the outset, it is essential to understand the role of Mg in our bodies. Mg is a vital nutrient required for various physiological functions, including nerve and muscle function, DNA synthesis, and protein synthesis. It is also a critical component of the immune system, helping to regulate inflammation and support immune cell function. Recent studies have indicated a direct link between low serum Mg levels and increased severity of COVID-19 symptoms. Low Mg levels have been linked to increased inflammation, decreased immune function, increased oxidative stress and cytokine storm, and poor oxygenation, which can contribute to worse COVID-19 outcomes. Moreover, low Mg levels may also contribute to the development of disseminated intravascular coagulation (DIC) through immune dysregulation, cytokine storm, and, ultimately, endothelial dysfunction in COVID-19 patients [13-16]. The clinical spectrum of SARS-CoV-2 infection tends to be very wide, ranging from asymptomatic infection, minor illness, and moderate upper respiratory tract disease to viral severe pneumonia with respiratory failure and death [17].

Given the critical role of Mg, it is not surprising that its deficiency can be associated with significant health implications. This led us to consider the potential therapeutic benefits of Mg supplementation in COVID-19 treatment when appropriate, which may influence immune resilience and patient morbidity and mortality [18]. However, it is essential to note that excessive Mg supplementation can have several adverse effects, including nausea, diarrhea, and stomach cramps. In severe cases, it can even lead to low blood pressure and irregular heart rhythm [19].

This systematic review aimed to (1) evaluate the association between serum Mg levels and COVID-19 severity and outcomes and (2) assess the potential therapeutic benefits of Mg supplementation in COVID-19 patients. We hypothesized that lower serum Mg levels would correlate with worse COVID-19 outcomes and that Mg supplementation could provide therapeutic benefits.

## Review

Materials and methods

Data Sources and Search Strategies

This systematic review was carried out per the guidelines of the Preferred Reporting Items for Systematic Reviews and Meta-Analyses (PRISMA) statement and registered in OSF (Open Science Framework) [20]. The literature search was conducted from January 1, 2020, to February 28, 2023, covering the period from the emergence of COVID-19 through our analysis cutoff date, and completed within two weeks. We used specific keywords: ("magnesium or low magnesium or hypomagnesemia or magnesium deficiency or micronutrients or trace minerals or electrolyte imbalance or malnutrition") and ("COVID-19 or SARS COV 2 or coronavirus") and ("outcome or role or associations or benefit or therapeutic benefit or supplement or effects"). Several databases, including MEDLINE (via PubMed), SCOPUS, Web of Science, Cochrane Library, and EMBASE, were searched using a thorough search approach. The search was done without any geographic location limits to capture all relevant titles.

Study Selection

All relevant articles were merged into a single file. After completing the database search, we employed Rayyan QCRI software to eliminate duplicate entries. Two reviewers independently screened the titles and abstracts of the remaining articles; a third reviewer resolved any disagreements between the two reviewers. Appropriate studies were collected for full-text screening; non-relevant articles were removed. Four reviewers working in pairs conducted the full-text screening of each downloaded article based on our inclusion criteria. Reasons for exclusion were reported; we used the sequential exclusion method and prioritization to reduce subjectivity in reporting the reasons for exclusions.

Eligibility Criteria

Studies examining the correlation between decreased serum Mg levels and COVID-19 outcomes and studies focusing on the potential therapeutic benefits of Mg supplementation in COVID-19 patients were considered for the systematic review. Case reports, review papers, opinion papers, editorials, commentary, and letters of editors were excluded. The inclusion and exclusion criteria of our study are summarized in Table 1.

**Table 1:** Eligibility criteria COVID-19: coronavirus disease 2019; Mg: magnesium

Inclusion criteria	Exclusion criteria
Studies that focus on the association between reduced serum Mg levels and COVID-19 outcomes	Studies that do not include both patients with confirmed COVID-19 and the effect of Mg levels on outcomes
Studies that explore the potential therapeutic benefits of Mg supplementation in COVID-19 patients	Case reports, case series, reviews, viewpoints, opinion papers, editorials, commentaries, letters to the editors, etc.
People of all ages, gender, ethnicity, and geographic location should be included	Studies published in languages other than English
Original research studies, including randomized controlled trials, non-randomized trials, analytic studies, observational studies, and case-control studies	All animal studies such as studies on rats, mice, rabbits, etc
Studies published in English	Unpublished studies

Data Extraction

Data extraction was done based on author, publication year, study design, period of study, geographical location of the study, population, participants, age, sex and race, comorbidities, baseline Mg level, Mg supplementation (doses and follow-up), p-value, 95% confidence interval (CI), COVID-19 severity (mortality, ICU stay, etc.), and length of hospital stay.

Risk of Bias Assessment

Two independent reviewers assessed the risk of bias using the RoBANs tool (Risk of Bias Assessment Tool for Non-randomized Studies) [21]. The lead researcher resolved any disagreement between the reviewers.

Data Analysis

Extracted data were examined to see if hypomagnesemia is associated with a worse outcome in COVID-19 and if it increases mortality; the potential causes of hypomagnesemia in COVID-19 patients; and if Mg supplementation has any therapeutic benefit in COVID-19 patients. Overall, demographics of people of all ages, genders, ethnicities, and geographic locations with laboratory-confirmed COVID-19 were reviewed. The association between Mg levels and COVID-19 outcomes was presented as relative risk (RR) and odds ratio (OR) with a 95% CI for dichotomous data [1].

Results

**Figure 1:**
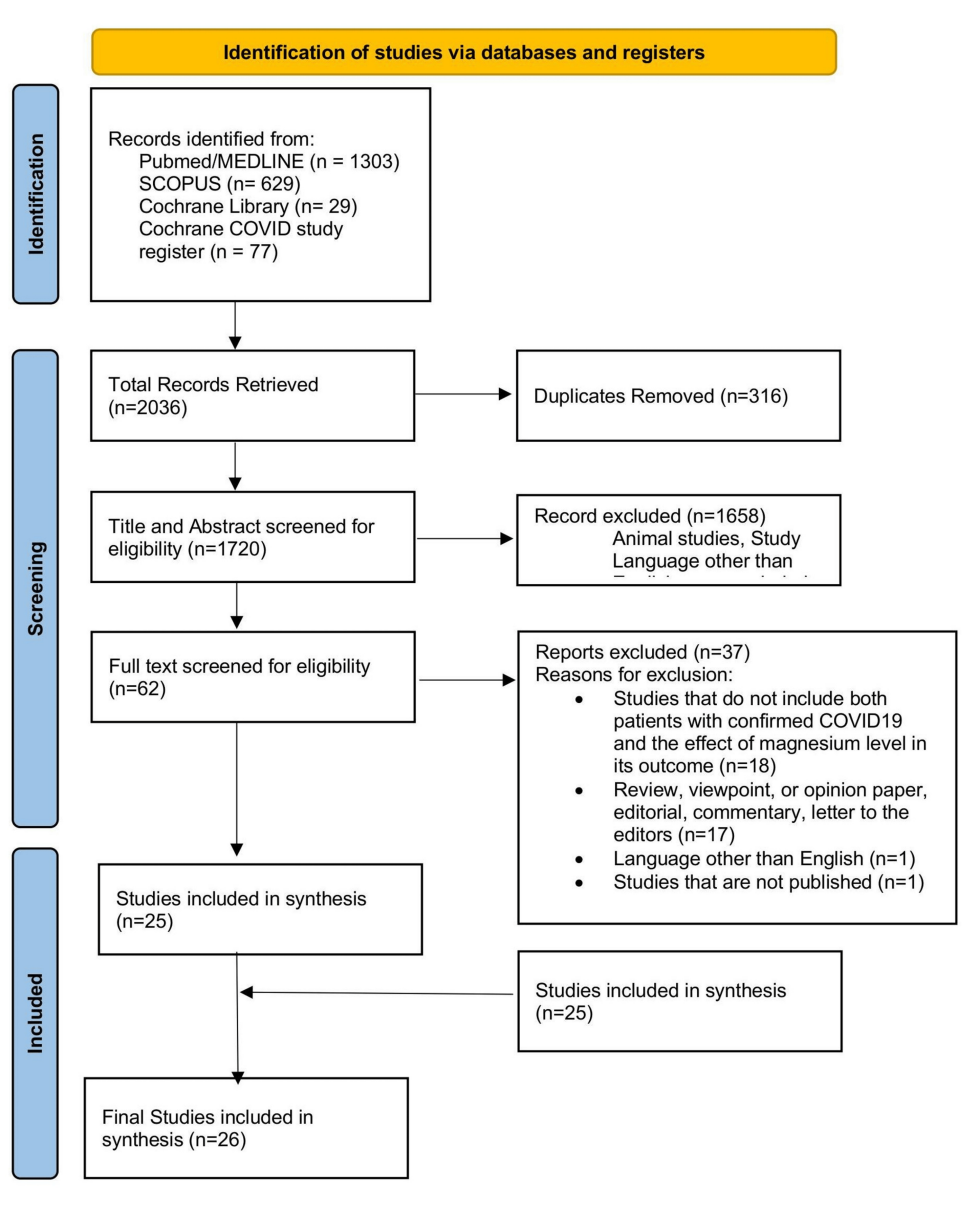
Flow diagram illustrating the study selection process

The review process details are depicted in the PRISMA flowchart in Figure 1. A total of 2033 records were identified. After removing duplicates, 1720 records remained and were screened for pertinent content. Of these, 1658 were excluded based on the title and abstract, leaving 62 studies to be assessed for eligibility. Reasons for exclusion were as follows: language other than English (n=1); studies that did not include both patients with confirmed COVID-19 and the effect of Mg level in its outcome (n=18); review, viewpoint, or opinion paper, editorial, commentary, letter to the editors (n=17); and unpublished studies (n=1). Finally, 26 studies met the criteria and were included in this review. Among them, 22 were related to serum Mg level and its relation to the severity of COVID-19. The other four dealt with Mg supplementation and its impact on COVID-19. The total number of participants was 11,363. Four studies focused on Mg supplementation and COVID-19 severity, encompassing 2889 participants. Other 22 studies focused on serum Mg levels and their impact on COVID-19, including 8474 participants.

Participants and Study Characteristics

The study included 26 studies: 11 cross-sectional studies, five case-control studies, eight retrospective cohort studies, and two prospective cohort studies. The studies encompassed a wide range of age groups (17-99 years). Some studies specified a mean or median age, while others provided a range, and three studies did not specify the age group; 16 studies had both male and female participants, while four studies focused solely on females and five solely on males; sex representation was not specified in one study. The baseline characteristics of the 26 included articles are summarized in Table 2.

**Table 2:** Key characteristics of studies included in the systematic review AKI: acute kidney injury; CKD: chronic kidney disease; COVID-19: coronavirus disease 2019; ICU: intensive care unit; Mg: magnesium; PTH: parathyroid hormone; RT-PCR: reverse transcription-polymerase chain reaction; TCZ: tocilizumab

Serial no.	Author	Study period	Study design	Geographic location	Population	Participant age, years	Participant sex
1	Anuk et al. [22]	May 2020 and August 2020	Case-control study	Ankara City Hospital, Turkey	200 (control: 100, case: 100)	17–38	Female
2	Zhu et al. [23]	January 27 to March 10, 2020	Retrospective cohort study	Guanggu Hospital District, Wuhan Third Hospital, China	83	Median age: 64	Not mentioned
3	Yasari et al. [24]	March 6 to the end of June 2020	Cross-sectional study	Masih Daneshvari Hospital, Tehran, Iran	169 hospitalized patients with COVID-19	Mean age: 53	Male and female
4	Al-Jassas et al. [25]	September to November 2020	Case-control study	Al-Sadr Teaching Hospital and Al-Amal Specialized Hospital for Communicable Diseases in Najaf Governorate, Iraq	60 COVID-19 patients and 30 healthy controls	25-59	Male only
5	Miri et al. [26]	May to August 2020	Retrospective cross-sectional study	Imam Reza Hospital, Mashhad, Iran	564	Mean age: 58.87 ± 1.82	Male (60%)
6	Haroon [27]	August to December 2020	Retrospective cohort study	OMI Hospital and Dr. Ziauddin Hospital, Karachi, Pakistan	102 ICU-admitted patients	Mean age: 63.2 ± 13.2.57; 8% were above 60 years, 37.3% were between 41-60 years, and the rest were below 40 and above 18 years	Male and female
7	Beigmohammadi et al. [28]	January 27 to March 10, 2020	Cross-sectional study	Imam Khomeini Hospital, Tehran, Iran	60 patients with COVID-19 who were admitted to the ICU	>20	Male and female
8	Duksal et al. [29]	May 2020 and January 2021	Retrospective cross-sectional study	Konya Tertiary Care Hospital, Konya, Turkey	522 [392 patients with TCZ application (case) and 130 patients without TCZ (control)]	Mean age: 62.0 ± 15.6	Male (64.4%)
9	Pulido Perez et al. [30]	March 24, 2020 to March 22, 2022	Retrospective cohort study	University Hospital of Peubla, Puebla, Mexico	390 adult patients diagnosed with COVID-19 and hospitalized	27-99	Male and female (238 men and 152 women)
10	Rostami et al. [31]	Not specified	Retrospective cohort study	Bakiattollah Hospital, Tehran, Iran	300 hospitalized patients due to COVID-19	Average age: 58.2	Male and female
11	Cozzolino et al. [32]	December 15, 2020 to May 15, 2021	Prospective observational (case-control) study	COVID Center at the University of Campania Luigi Vanvitelli, Naples, Italy	208	26-94	Male (61.5%)
12	Chau et al. [33]	Not specified	Retrospective cohort study	Not specified	139 adult COVID-19 patients on respiratory support into less than 24 hours of admission	Not specified	Male and female
13	Abdulla and Abed [34]	September 2021 to August 2022	Case-control study	Samarra Hospital, Samarra, Iraq	105 females including 30 healthy individuals and 75 confirmed cases of COVID-19	20-38	Female
14	Tanacan et al. [35]	March 11 to October 30, 2020	Prospective cohort study	Turkish Ministry of Health Ankara City Hospital, Ankara, Turkey	133	Not mentioned	Female
15	Sabaghian et al. [36]	February 2021 to March 2021	Retrospective cohort study	Imam Hossein Hospital, a tertiary healthcare center in Tehran, Iran	567 patients with positive COVID-19 RT-PCR test; 499 hospitalized patients with confirmed COVID-19 without CKD; AKI group: 168 patients, non-AKI group: 331 patients	AKI mean age: 67; non-AKI mean age: 56	Male and female
16	Guerrero-Romero et al. [37]	March 2020 to July 2021	Retrospective cross-sectional study	A tertiary care hospital in Mexico City and a general hospital in Durango City in central and northern Mexico, respectively	Screening patients hospitalized with COVID-19: 1616; enrolled patients: 1064; discharged per death: 554; discharged per recovery: 510	Mean age: 60.3 ± 15.7	Male and female
17	Hashemipour et al. [38]	January to April 2021	Prospective cross-sectional study	Booali Education and Therapeutic Center, Qazvin Province, Iran	123 hospitalized patients with COVID-19; serum concentration of PTH, 25(OH)D, Mg, phosphate, and albumin were assessed in comparison with moderate/severe, mild, and normal calcemia	>18 years	Male and female
18	Díez et al. [39]	January 1, 2020, to January 1, 2021	Retrospective analytical study	Hospital Universitario Puerta de Hierro Majadahonda (HUPHM), Madrid, Spain	2473 COVID-19 patients	Mean age: 63.4 ± 15.9	Male and female
19	Bonakdaran et al. [40]	Not specified	Cross-sectional study	Emergency department of the Imam Reza Hospital, Mashhad, Iran	120 patients: 70 patients were analyzed; 50 patients were excluded due to a lack of accurate documents	Mean age: 60.1 ± 15.3	Male and female
20	Al-Hakeim et al. [41]	September to November 2020	Case-control study	Al-Sadr Teaching Hospital and Al Amal Specialized Hospital for Communicable Diseases, Najaf Governorate, Iraq	60 COVID-19 patients and 30 healthy controls	25-59	Male
21	Kiran Kumar et al. [42]	Not specified	Cross-sectional comparative study	All India Institute Of Medical Sciences, Jodhpur, India	150 COVID-19 patients and 50 healthy individuals	Not specified	Male and female
22	Quilliot et al. [43]	March 1, 2020, to April 29, 2020	Prospective cohort Study	Nancy Brabois University Hospital, Vandœuvre-lès-Nancy, France	300 hospital-admitted COVID-19 patients	>18	Male and female (male: 183, female: 117)
23	Citu et al. [44]	April 2020 to February 2022	Cross-sectional study	Timișoara Municipal Emergency Hospital, Timisoara, Romania	448 pregnant females	All age groups	Female
24	Nimer et al. [45]	March to July 2021	Cross-sectional study	Jordan University of Science and Technology, Irbid, Jordan	2148 individuals who recovered from COVID-19 disease	24-56	Male and female
25	Nouri-Majd et al. [46]	June to September 2021	Retrospective cross-sectional study	Shahid Beheshti Hospital, Kashan, Iran	250	18-65	Male and female
26	Tan, C.W. et al. [47]	January 15, 2020, to April 15, 2020	Retrospective cohort study	Singapore General Hospital, Singapore	53	50 and above	Male and female

Risk of Bias Assessment

**Figure 2:**
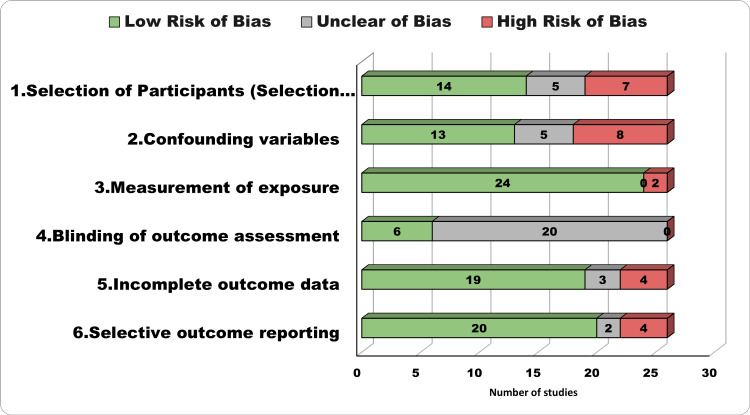
Risk of Bias Assessment Tool for Non-randomized Studies (RoBANS)

We thoroughly assessed the risk of bias in the 26 studies in our review, employing the RoBANs tool across six key domains as illustrated in Figure 2. These domains encompassed participant selection, management of confounding variables, accuracy of exposure measurement, blinding of outcome assessments, incomplete outcome data, and transparency in outcome reporting. The evaluation of bias yielded a mixed picture. Quality and risk of bias varied across the included studies. Notably, exposure measurement was a strong point, with most studies (24/26) demonstrating a low risk of bias. Conversely, blinding of outcome assessment posed a significant concern, as none of the studies achieved a low risk of bias in this aspect, with most having an unclear risk of bias (20 studies). Confounding variables and incomplete outcome data also showed variability. At the same time, a majority demonstrated low risk in controlling confounding variables (13 studies) and handling incomplete outcome data (19 studies). A notable portion exhibited high risk in both domains (eight studies for confounding variables, four for incomplete outcome data). Selective outcome reporting appeared to be predominantly low risk, with 20 studies; however, caution is warranted for the four studies categorized as high risk.

COVID-19 Severity Assessment 

Various methods were employed to assess COVID-19 severity in the studies. These methods included clinical criteria such as respiratory rate, oxygen saturation, and symptoms [22-27]; the Acute Physiology and Chronic Health Evaluation (APACHE) score to gauge severity [28]; ICU admission [29-31]; the presence of arrhythmia [32]; BMI categorization [30]; need for mechanical ventilation [33,27]; chest CT abnormalities [25]; clinical stages [34]; obstetric complications [35]; CT scores [26]; and acute kidney injury [36].

Association Between COVID-19 Severity and Serum Magnesium Levels 

Twenty-two studies involving 8474 patients that assessed serum Mg levels' effect on COVID-19 severity outcomes found mixed results. The majority of studies demonstrated that patients with severe COVID-19 had significantly lower serum Mg levels compared to those with mild or moderate disease. These lower Mg levels were consistently associated with poorer clinical outcomes. In Zhu et al.'s study, serum Mg was significantly decreased in the critical group compared with the other groups (p<0.05) [23]. Similarly, Beigmohammadi et al. reported that lower serum Mg levels were significantly associated with higher APACHE scores (p=0.028) [28]. Yasari et al. also found that the median serum Mg level was considerably lower in patients with critical disease than those with moderate and severe disease (p<0.001) [24]. Al-Jassas et al. reported that COVID-19 patients with SpO_2_ values <76% had significantly lower Mg levels compared to healthy controls and COVID-19 patients with SpO_2_ values ≥76% (p<0.001) [25].

Guerrero-Romero et al. found that the proportion of hypomagnesemia was significantly higher in patients discharged per death than in those discharged per recovery (p=0.002) [37]. Additionally, a magnesium-to-calcium ratio ≤0.20 was strongly associated with mortality in severe COVID-19 (p=0.01) [37]. However, in univariate and multivariate analysis, Hashemipour et al. found that Mg deficiency was not associated with hypocalcemia in COVID-19 patients (p=0.553) [38]. Díez et al. initially suggested that elevated Mg levels were related to a poor outcome, but this significant relationship was not maintained in the multivariate analysis (p=0.001) [39]. Bonakdaran et al. also found no association (p=0.181) [40]. However, Sabaghian et al. reported that hypermagnesemia, either on admission or during hospitalization, was associated with a higher death rate in COVID-19 patients with acute kidney injury (AKI) (p<0.05) [36]. Tanacan et al. found that hypermagnesemia positively correlated with disease severity and obstetric complications [35]. Furthermore, a negative correlation was observed between hypomagnesemia and obstetric complications (p=0.03).

Duksal et al. reported that the rate of hypomagnesemia was low in both cases (3.2%) and controls (3.8%) [29]. Conversely, hypermagnesemia was found to be higher than hypomagnesemia. The rate of hypermagnesemia was found to be 26.7% in cases, while it was higher (30.9%) in patients treated in the ICU (p<0.001). Cozzolino et al. found that serum Mg levels in patients with arrhythmia were lower than those without (p=0.04) [32]. Miri et al. reported that patients with poor prognoses based on CT scores had significantly higher serum Mg (2.27 ± 0.39 mg/dL) (p=0.035) than patients with good prognoses (2.10 mg/dL) [26]. Abdulla and Abed found that Mg concentrations were decreased in patient groups compared with controls [34]. Additionally, reduced Mg level was associated with increased severity (p=0.0001).

Pulido Perez et al. reported that lower Mg levels were associated with increased mortality in obese COVID-19 patients (p<0.0001) [30]. Rostami et al. (p=0.003) and Al-Hakeim et al. (p=0.002) observed lower Mg levels in COVID-19 patients compared to healthy controls. They also suggested a trend of even lower Mg levels in patients with more severe illness, as measured by ICU admission or abnormal chest CT scans [31,41]. Kiran Kumar et al. (p=0.7475) found no significant difference in Mg levels between COVID-19 patients and healthy individuals, regardless of disease severity [42]. Interestingly, Haroon (p=0.19) even observed a higher prevalence of high Mg levels in patients with severe COVID-19, although this finding was not statistically significant [27]. Adding to the complexity, Didier et al. reported a high prevalence of low Mg across all hospitalized COVID-19 patients in their study (p<0.001), regardless of disease severity [43]. A summary of these findings is presented in Table 3.

**Table 3:** Association between COVID-19 and serum Mg levels AKI: acute kidney injury; APACHE: Acute Physiology and Chronic Health Evaluation; CKD: chronic kidney disease; COVID-19: coronavirus disease 2019; CT: computed tomography; ICU: intensive care unit; Mg: magnesium; PTH: parathyroid hormone; RT-PCR: reverse transcription-polymerase chain reaction; TCZ: tocilizumab

Serial no.	Author	Population	Categorization	Findings	Statistical significance
1	Anuk et al. [22]	200 (control: 100, case: 100)	COVID-19 group: first trimester (n=34), second trimester (n=33), third trimester (n=33); control group: first trimester (n=33), second trimester (n=32), third trimester (n=35)	COVID-19 in the first and third trimesters, serum Mg levels increased compared to controls	P<0.0001
2	Zhu et al. [23]	83	Moderate (n=25), severe (n=26), and critical (n=32) disease	Serum Mg was significantly decreased in the critical group compared with the other groups	P<0.05
3	Beigmohammadi et al. [28]	60	APACHE score 25 (n=20) and APACHE score <25 (n=40). A high APACHE score predicts the severity of COVID-19 and increased risk of mortality	Lower serum levels of Mg are significantly associated with higher APACHE scores	P=0.028
4	Yasari et al. [24]	169	Moderate (n=37), severe (n=80), and critical (n=52) disease	Median serum Mg level was significantly lower in patients with critical disease compared to those with moderate disease and severe disease	p < 0.001
5	Al-Jassas et al. [25]	60 COVID-19 patients and 30 healthy controls	Healthy controls (n=30), COVID-19 with SpO_2_ ≥76% (n=33), COVID-19 with SpO_2_ <76% (n=27)	COVID-19 patients with SpO_2_ values <76% had significantly lower Mg levels compared to healthy controls and COVID-19 patients with SpO_2_ values ≥76%	P<0.001)
6	Guerrero-Romero et al. [37]	1616	Total screening patients hospitalized due to COVID-19: 1616; enrolled patients: 1064; discharged per death: 554; discharged per recovery: 510	The proportion of hypomagnesemia (54.3% vs. 32.9%, p=0.002) and hypocalcemia (16.4% vs. 9.5%, p=0.01) was significantly higher in the patients discharged per death than in those discharged per recovery. Additionally, magnesium-to-calcium ratio ≤0.20 is strongly associated with mortality in severe COVID-19	P=0.01
7	Hashemipour et al. [38]	123	123 hospitalized patients with COVID-19; serum concentration of PTH, 25(OH)D, Mg, phosphate, and albumin were assessed in comparison with moderate/severe, mild, and normal calcemia	Mg deficiency was not associated with hypocalcemia in COVID-19 patients in univariate and multivariate analyses	P=0.553
8	Díez JJ et al. [39]	2473	2473 COVID-19 patients	Initial analysis suggested that elevated Mg levels were related to poor outcomes. However, in the multivariate analysis, this significant relationship was not maintained	P=0.001
9	Bonakdaran et al. [40]	120	120 patients: 70 patients were analyzed; 50 patients were excluded due to a lack of accurate documents	No association is found	P=0.181
10	Sabaghian et al. [36]	567	567 patients with positive COVID-19 RT-PCR test; 499 hospitalized patients with confirmed COVID-19 without CKD; AKI group: 168 patients, non-AKI group: 331 patients	Hypermagnesemia either on admission or during hospitalization is associated with a higher death rate in COVID-19 patients with AKI	P<0.05
11	Tanacan et al. [35]	133	Mild COVID-19 group (n=811) and moderate/severe COVID-19 group (n=52)	Hypermagnesemia was found to be positively correlated with both disease severity and obstetric complications. Furthermore, a negative correlation was observed between hypomagnesemia and obstetric complications	P=0.03
12	Duksal et al. [29]	522	522 [392 patients with TCZ application (case) and 130 patients without TCZ (control)]. Cases further divided into wards (n=164) and ICU (n=228)	The rate of hypomagnesemia was low in both cases (3.2%) and controls (3.8%). Conversely, hypermagnesemia was found to be higher than hypomagnesemia. The rate of hypermagnesemia was found to be 26.7% in cases while it was higher (30.9%) in patients treated in the ICU	P<0.001
13	Cozzolino et al. [32]	208	Patients without arrhythmia: 120 (I60%); patients with arrhythmia: 80 (40%)	Serum Mg levels in patients with arrhythmia were lower than those in patients without arrhythmia	P=0.04
14	Miri et al. [26]	564	Severe and non-severe COVID-19 patients based on the WHO criteria, (a respiratory rate above 30/min or oxygen saturation below 93% are the criteria for severe COVID-19); good and poor prognosis based on the CT score	Patients with poor prognosis based on CT scores had significantly higher serum Mg (2.27 ± 0.39 mg/dL) (p=0.035) than patients with good prognosis (2.10 mg/dL)	P=0.035
15	Abdulla and Abed [34]	105 females including 30 healthy individuals and 75 confirmed cases of COVID-19	COVID-19 patients were divided into three groups and were categorized according to the clinical stages with each including 25 patients: stage I, stage II, and stage III	Mg concentrations were decreased in patient groups compared with controls. Additionally, decreased Mg was associated with increased severity	P=0.0001
16	Pulido Perez et al. [30]	390 adult patients diagnosed with COVID-19 and hospitalized	Categorization according to BMI: normal (n=95), overweight (n=160), and obese (n=135)	Lower Mg levels were associated with increased mortality in obese COVID-19 patients	P<0.0001
17	Rostami et al. [31]	300 hospitalized patients due to COVID-19	Categorization: ICU patients (n=132); non-ICU in-patients (n=168)	Hypomagnesemia is a common finding in COVID-19 patients and is more common in severe cases	P=0.003
18	Chau et al. [33]	139 adult COVID-19 patients on respiratory support into less than 24 hours of admission	Adult COVID-19 patients on respiratory support in less than 24 hours of admission (n=139)	Critically ill COVID-19 patients experienced more severe electrolyte abnormality and required more electrolyte repletion than non-critically ill patients	
19	Haroon [27]	102 ICU-admitted patients	Requiring mechanical ventilation or not, survival	Patients with severe COVID-19. Hypermagnesemia was more common than hypermagnesemia but no statistical difference between different severity levels	P=0.19
20	Al-Hakeim et al. [41]	60 COVID-19 patients and 30 healthy controls	Healthy controls (n=30); COVID-19, no chest CT abnormality (n=17); COVID-19 + chest CT abnormality (n=43)	Mg levels are lower in COVID-19 patients compared to healthy controls and maybe even lower in those with chest CT abnormalities	P=0.002
21	Kiran Kumar et al. [42]	150 COVID-19 patients and 50 healthy individuals	150 COVID-19 patients were divided into mild, moderate, and severe groups with each group having 50 individuals. Another 50 individuals were included as healthy controls	No significant change in the levels of serum magnesium between controls and cases as well as between the different severity groups of cases	P=0.7475
22	Quilliot et al. [43]	300 hospital-admitted COVID-19 patients	Moderate (n=43), severe (n=108), and critical (n=149) disease	This transversal study revealed a high prevalence of hypomagnesemia in 300 patients hospitalized for COVID-19	P<0.001

Magnesium Supplementation and COVID-19 Outcomes

Four studies assessed the effect of Mg supplements on COVID-19 severity. Citu et al.'s study involving 448 pregnant females found that Mg supplementation, particularly when it contained more than 450 mg of Mg, was associated with higher antibody titers after COVID-19 (p=0.044) [44]. Nimer et al.'s study of 2148 individuals who recovered from COVID-19 found no significant association between Mg intake and COVID-19 severity or hospitalization (p=0.73 for severity, p=0.24 for hospitalization) [45]. In contrast, Nouri-Majd S's study of 250 COVID-19 hospitalized patients demonstrated that higher dietary Mg intake was linked to a shorter hospitalization duration, faster recovery, a reduced chance of experiencing COVID-19 symptoms, and lower inflammatory biomarker levels (p<0.001) [46]. Lastly, Tan et al. conducted a study with 43 participants and observed that a combination of vitamin D, Mg, and vitamin B12 supplementation in older COVID-19 patients was associated with a significant decrease in the proportion of patients needing oxygen or ICU support (p=0.006) [47]. The details of these outcomes are presented in Table 4.

**Table 4:** Magnesium supplementation and its effect on COVID-19 Ca: calcium; CRP: C-reactive protein; COVID-19: coronavirus disease 2019; ESR: erythrocyte sedimentation rate; Mg: magnesium; Zn: zinc

Serial no.	Author	Population	Categorization	Outcome	Statistical significance
1	Citu et al. [44]	448 pregnant females	No supplementation (n=313), Mg supplementation (n=61), Ca+Mg+Zn supplementation (n=74)	Mg >450 mg in the taken supplements determined higher levels of antibody titers after COVID-19	P=0.044
2	Nimer et al. [45]	2148 individuals who recovered from COVID-19 disease	Not mentioned	Logistic regression analysis indicated no association between Mg intake and COVID-19 severity and hospitalization	P=0.73 for severity. P=0.24 for hospitalization
3	Nouri-Majd et al. [46]	250	250 COVID-19 hospitalized patients. Participants were selected from a group of improved COVID-19 patients who had been initially diagnosed before a maximum duration of 3 months	Higher Mg intake was associated with a shorter duration of hospitalization and convalescence, as well as a lower chance of having COVID-19 symptoms. Additionally, a higher dietary magnesium intake was associated with lower inflammatory biomarker concentrations (CRP and ESR)	P<0.001
4	Tan et al. [47]	43	Supplement with vitamin D, Mg, vitamin B12 (n=17); controls (n=26)	Vitamin D/Mg/vitamin B12 combination in older COVID-19 patients was associated with a significant reduction in the proportion of patients with clinical deterioration requiring oxygen support and/or intensive care support	P=0.006

Discussion

Summary of Findings

This systematic review provides novel insights into the literature on Mg's role in COVID-19. Our analysis is the first to simultaneously examine serum Mg levels (22 studies, n=8,474) and supplementation effects (four studies, n=2,889) in a large, combined sample (11,363 patients). We aimed to find whether serum Mg levels or the use of Mg supplements affected the outcome of COVID-19 infections. The review explored diverse populations and infection severities. Lower Mg levels were associated with increased severity of COVID-19, as observed in multiple studies. Notably, lower Mg levels were found in patients with critical disease than in patients with moderate or severe conditions. Mg supplementation, particularly doses over 450 mg, showed potential benefits, such as increased antibody titers and reduced need for oxygen assistance in elderly patients.

Moreover, dietary Mg intake was linked to shorter hospital stays and recovery times. However, conflicting findings were noted, with some studies failing to establish a direct correlation between Mg levels and COVID-19 severity or mortality. While specific investigations identified genetic predispositions to lower Mg concentrations in COVID-19 cases, others found no significant impact of Mg supplementation on disease severity or hospitalization risk. In summary, the collective evidence suggests a potential association between Mg levels and COVID-19 outcomes, emphasizing the need for further research to elucidate the complexities of this relationship.

Agreement and Disagreement With Contemporary Research

We found several studies that observed that serum Mg abnormalities are associated with adverse outcomes in COVID-19 patients. Some refer to the positive therapeutic effect of Mg supplementation. Recent studies have provided further insights into the relationship between Mg levels and COVID-19 outcomes. A randomized controlled trial found that Mg supplementation during COVID-19 treatment reduced the likelihood of patients requiring oxygen therapy [48]. This aligns with earlier research suggesting that higher dietary Mg intake is inversely associated with COVID-19 severity and symptoms [49]. However, some studies have reported conflicting results, with both hypomagnesemia and hypermagnesemia being associated with increased mortality in COVID-19 patients [50].

In individuals with low Mg levels, there appears to be a heightened susceptibility to COVID-19, as suggested by various studies. Low Mg status is linked to increased infection risk, unfavorable prognosis in hospitalized patients, and neuropsychiatric complications associated with COVID-19. Moreover, Mg intake, both through diet and pulmonary inhalation, demonstrates potential protective effects and improved oxygenation. Additionally, Mg and zinc may enhance the efficacy of COVID-19 therapies or mitigate their side effects, as discussed in relevant reviews [18]. Insufficient calcium, phosphorus, and Mg levels can compromise immune function, potentially elevating vulnerability to viral infections, including COVID-19 [51]. Males were diagnosed concurrently with hypermagnesemia and were at a greater risk of mortality. However, the authors recommended further research to understand the underlying pathophysiology [52]. Upon patient admission, identifying low serum Mg levels can be a cost-effective biomarker for adverse outcomes in COVID-19 patients and help clinicians stratify risk. However, further research is essential [53]. On the other hand, other authors concluded that low serum Mg levels could not be used to predict COVID-19 prognosis and that hypermagnesemia might be associated with COVID-19 mortality [54].

Implication of Findings

Despite suggestive evidence pointing towards a possible association between low serum Mg levels and worse COVID-19 outcomes, the inconclusiveness of available data emphasizes the need for further comprehensive research. Understanding the role of Mg in the pathophysiology of COVID-19 could guide the development of targeted therapeutic strategies for managing and treating COVID-19 patients. Continued investigation is crucial to clarify the relationship between Mg levels, its supplementation, and COVID-19 outcomes.

Limitations of the Study

It is important to emphasize that this systematic study has a few limitations. Various study designs, sample sizes, and demographic characteristics of the included studies could have made the results heterogeneous, making a meta-analysis impossible. In addition, many of the studies were observational, making it challenging to prove any causation. The inability of trials to compare findings due to the lack of standardized procedures for assessing serum Mg levels should also be taken into account.

## Conclusions

The findings of this comprehensive investigation point to a possible association between low serum Mg levels and the worsening severity and outcomes of COVID-19, including higher disease severity, increased mortality rates, and prolonged recovery periods. Further research is strongly recommended as a better understanding of the role of Mg in the etiology of COVID-19 may help develop targeted medicines and therapeutic methods for managing COVID-19 patients.
